# Pathological Neuroinflammatory Conversion of Reactive Astrocytes Is Induced by Microglia and Involves Chromatin Remodeling

**DOI:** 10.3389/fphar.2021.689346

**Published:** 2021-06-21

**Authors:** Alejandro Villarreal, Camila Vidos, Matías Monteverde Busso, María Belén Cieri, Alberto Javier Ramos

**Affiliations:** ^1^ Laboratorio de Neuropatología Molecular, Instituto de Biología Celular y Neurociencia “Prof. E. De Robertis” UBA-CONICET, Facultad de Medicina, Universidad de Buenos Aires, Buenos Aires, Argentina; ^2^ Primera Unidad Académica de Histología, Embriología, Biología Celular y Genética, Facultad de Medicina, Universidad de Buenos Aires, Buenos Aires, Argentina

**Keywords:** neuroinflammation, reactive astrogliosis, epigenetics, NF-κB, microglia–astrocyte crosstalk

## Abstract

Following brain injury or in neurodegenerative diseases, astrocytes become reactive and may suffer pathological remodeling, features of which are the loss of their homeostatic functions and a pro-inflammatory gain of function that facilitates neurodegeneration. Pharmacological intervention to modulate this astroglial response and neuroinflammation is an interesting new therapeutic research strategy, but it still requires a deeper understanding of the underlying cellular and molecular mechanisms of the phenomenon. Based on the known microglial–astroglial interaction, the prominent role of the nuclear factor kappa B (NF-κB) pathway in mediating astroglial pathological pro-inflammatory gain of function, and its ability to recruit chromatin-remodeling enzymes, we first explored the microglial role in the initiation of astroglial pro-inflammatory conversion and then monitored the progression of epigenetic changes in the astrocytic chromatin. Different configurations of primary glial culture were used to modulate microglia–astrocyte crosstalk while inducing pro-inflammatory gain of function by lipopolysaccharide (LPS) exposure. *In vivo*, brain ischemia by cortical devascularization (pial disruption) was performed to verify the presence of epigenetic marks in reactive astrocytes. Our results showed that 1) microglia is required to initiate the pathological conversion of astrocytes by triggering the NF-κB signaling pathway; 2) this interaction is mediated by soluble factors and induces stable astroglial phenotypic changes; 3) the pathological conversion promotes chromatin remodeling with stable increase in H3K9K14ac, temporary increase in H3K27ac, and temporary reduction in heterochromatin mark H3K9me3; and 4) *in vivo* reactive astrocytes show increased H3K27ac mark in the neuroinflammatory milieu from the ischemic penumbra. Our findings indicate that astroglial pathological pro-inflammatory gain of function is associated with profound changes in the configuration of astrocytic chromatin, which in turn are initiated by microglia-derived cues. These results open a new avenue in the study of potential pharmacological interventions that modify the initiation and stabilization of astroglial pathological remodeling, which would be useful in acute and chronic CNS injury. Epigenetic changes represent a plausible pharmacological target to interfere with the stabilization of the pathological astroglial phenotype.

## Introduction

Astrocytes are the main players in maintaining brain homeostasis, having cellular pathways that allow them to protect neurons from glutamate excitotoxicity, extracellular ion disbalances, ammonium accumulation, and reactive oxygen species, as has been extensively reviewed (Sofroniew and Vinters, 2010; Clarke and Barres, 2013; Pekny et al., 2016; Santello et al., 2019). Furthermore, astrocytes provide structural and metabolic support to neurons, modulate synapses, and take part in the blood–brain barrier, among several other beneficial functions on the healthy central nervous system (CNS).

Under pathological conditions such as traumatic brain injury and brain ischemia or in neurodegenerative diseases, astrocytes participate in a cellular response to damage known as reactive gliosis, along with microglia and other cell types. In particular, astrocytes suffer temporal and spatial gradual morphological alterations together with profound transcriptomic changes (Sofroniew, 2009; Sofroniew, 2015; Anderson et al., 2016). Morphological and molecular studies indicate that astrocyte response is heterogeneous across CNS regions and even within regions (Anderson et al., 2014; Ramos, 2016; Bribian et al., 2018; Matias et al., 2019).

Reactive astrogliosis is part of the nervous tissue response to injury, which is triggered as an attempt to control damage and promote regeneration (Barres, 2008; Cregg et al., 2014; Sofroniew, 2015). However, sustained pro-inflammatory signals may lead to astroglial pathological remodeling with a pro-inflammatory gain of function that exacerbates neuronal death (Liu et al., 2011, 2020; Lian et al., 2016; Kirkley et al., 2017; Liddelow et al., 2017; Verkhratsky et al., 2019; Matejuk and Ransohoff, 2020). Initiation of the reactive astrocyte pathological remodeling has been partially explained by previous work from our group and others. We have shown that DAMP (damage-associated molecular pattern) or PAMP (pathogen-associated molecular pattern) exposure induce astroglial pro-inflammatory and neurotoxic phenotype remodeling in a PRR (pattern recognition receptor)- and NF-κB–dependent manner (Angelo et al., 2014, 2014; Lian et al., 2015; Rosciszewski et al., 2017). However, the dynamics of the activation of these processes and the requirements of non–cell autonomous (i.e., extrinsic) components have not yet been fully addressed.

Once engaged in the pro-inflammatory gain of function, reactive astrocytes show a very stable phenotype (Ramos et al., 2004; Díaz-Amarilla et al., 2011; Sofroniew, 2015; Pekny et al., 2016; Villarreal et al., 2016). This intriguing stable phenotype is likely to be sustained by extrinsic (non–cell autonomous) or intrinsic (cell autonomous) mechanisms. Extrinsic mechanisms involve the constitutive release of soluble cues from neighbor cells such as microglia or other immunocompetent cell partners, which sustain a pro-inflammatory astrocyte microenvironment. On the other hand, the intrinsic mechanisms may involve long-term changes in gene expression, and thus, epigenetic mechanisms are the candidates for stabilization of the pro-inflammatory astroglial phenotype.

Specifically, it has been reported that NF-κB interacts with chromatin remodelers such as p300 acetyltransferase, recruiting them to specific regulatory regions and increasing local histone acetylation and transcription of pro-inflammatory genes (Berghe et al., 1999; Mukherjee et al., 2013). In general, histone acetylation correlates with active transcription. The enzymes responsible for adding acetyl groups are widely known as histone acetyl transferases (HATs). Their counterparts, responsible for removing acetyl groups, are named histone deacetylases (HDACs). The balance between the activities of these enzymes determines the levels of acetylated histones and active transcription. HDACs have already been investigated as possible targets to modulate inflammation (Grunstein, 1997; Marmorstein and Zhou, 2014; Tessarz and Kouzarides, 2014; Voss and Thomas, 2018). However, the molecular mechanisms regulated by these enzymes in neural cells, and specifically in astrocytes, are still far from being fully understood. Here, we explored global changes of histone acetylation in reactive astrocytes and their role in the stabilization of the pro-inflammatory astrocytic gain of function.

## Material and Methods

### Materials

Antibodies were purchased from Dako (rabbit polyclonal anti-GFAP, cat# Z0334), Abcam (goat polyclonal anti–Iba-1, cat# ab5076), Chemicon-Millipore (mouse monoclonal anti-Actin, cat# MAB1501), Sigma (mouse monoclonal anti-GFAP, cat# G3893), Santa Cruz (mouse monoclonal anti-p65, cat# sc-8008 and mouse monoclonal anti-H3K9K14ac, cat# sc518011), and Diagenode (H3K27AC, cat# C15410174 and H3K9me3, cat# C15410193, which were a kind gift from Prof. Dr Tanja Vogel, Institute for Anatomy and Cell Biology, Dept. of Molecular Embryology, University of Freiburg, Germany). Fluorescent secondary antibodies (Alexa Fluor 594 and 488) and peroxidase-conjugated secondary antibodies raised in donkey were purchased from Jackson Immunoresearch (United States).

Cell culture reagents were obtained from Invitrogen Life Technologies (Carlsbad, United States). Fetal calf serum (FCS) was purchased from Natocor (Córdoba, Argentina). Poly-L-lysine, DAPI (4′,6-diamidino-2-phenylindole), and other chemicals were purchased from Sigma (United States).

### Primary Cultures of Glial Cells

Primary cultures were obtained from postnatal day 2–3 cerebral cortex of mice as already described (Rosciszewski et al., 2019), with minor changes. Briefly, cortices from both hemispheres were dissected under a microscope and mechanically dissociated using tweezers and by pipetting up and down with a decreasing micropipette tip diameter. The supernatant was transferred to a new tube and centrifuged at 1000 rpm for 5 min to pellet the cells. The clear supernatant was removed, and the cells were resuspended in Dulbecco’s Modified Eagle Medium (DMEM) supplemented with 10% fetal calf serum, 2 mm L-glutamine, and 100 μg/ml penicillin/streptomycin (complete medium). The cells were plated in flasks and incubated at 37°C in a 5% CO_2_ atmosphere until confluence was reached, which typically takes one week. Medium change was conducted one day after plating and then after 2 or 3 days. After reaching confluence, flasks were treated according to each culture configuration. Untreated flasks lead to glial mixed cultures (GMC), which were split into poly-lysine–coated multiwell plates or single-well plates.

To obtain astrocyte-enriched culture (AEC), microglial depletion was achieved by 48 h of shaking under dark conditions, replacing the medium every 24 h. To eliminate proliferating microglia, 5-fluorouracyl (5-FU) was added to the medium and incubated for an additional 24 h. After 5-FU exposure, the astrocytes were washed 3 times with complete medium and incubated at least 24 h before splitting (Angelo et al., 2014).

Reconstituted glial cultures were performed with microglia collected from the supernatant 24 and 48 h after shaking under dark conditions. Cells were centrifuged, resuspended in fresh complete medium, and immediately added on the top of a monolayer of AEC. For experiments in which microglia-conditioned medium was required, microglia obtained as explained before was plated on poly-lysine–coated multiwell plates. Under every condition, the cells were incubated for at least 24 h before treatments.

### LPS Treatment

Cells from GMC, AEC, microglia-reconstituted AEC, and isolated microglia were replated at least 24 h before treatment. Two hours before treatment, complete medium was replaced with reduced-serum DMEM (2% FCS). LPS was added to each well at a final concentration of 25 ng/ml. This concentration was selected after dose-response studies that determined that GMC exposed to 25 ng/ml of LPS for 3 h promotes astroglial pro-inflammatory gain of function and neuronal death (Rosciszewski et al., 2019).

Using this protocol with 2% FCS, the untreated cells (namely, Ctrl) show a percentage of nuclear p65 in astrocytes that is below 0.5%. Microglia-conditioned medium was obtained by extracting medium after 1 h of exposure and immediately transferring it to wells containing AEC. Microglia was switched to complete medium until the end of the experiment and processed to confirm p65 nuclear translocation.

### Cortical Devascularization (Pial Disruption) Model of Ischemia

Adult male Wistar rats (300–350 g) were obtained from the Animal Facility of the National Atomic Energy Commission (Comisión Nacional de Energía Atómica, CNEA, Argentina). The animals were housed in a controlled environment (12/12-h light/dark cycle and controlled humidity and temperature, with free access to standard laboratory rat food and water) under the permanent supervision of professional staff. The animals were anesthetized with ketamine/xylazine (90/10 mg/kg i.p.) and subjected to a unilateral cortical devascularization (CD) as previously described (Herrera and Cuello, 1992; Villarreal et al., 2011; Villarreal et al., 2016), (Figure 6E). Briefly, the rats were placed in a stereotaxic apparatus, and a small surface of the skull between the coronal suture and the bregma line was removed to expose the underlying vasculature. A 27-gauge needle was used to cut the overlying dura and tear it away from the underlying pia. A sterile cotton swab was then used to tear back the pia and to disrupt the pial blood vessels overlaying the exposed cortex. Immediately, small sterile cotton pieces embedded in sterile saline solution were laid on the cortical surface until all bleeding ceased (usually less than 50 s). All cotton pieces were removed, and the incision in the overlying skin was then closed, using the temporal muscle and the attached fascia to cover the lesion site. After surgery, the animals were housed individually to allow recovery for 4 days. During the surgery and the whole awakening period, body temperature was maintained by means of a heating pad. Animal care for this experimental protocol was in accordance with NIH guidelines for the care and use of laboratory animals, the Guidelines for the Use of Animals in Neuroscience Research by the Society for Neuroscience, and the ARRIVE guidelines and was approved by the CICUAL Committee of the School of Medicine, University of Buenos Aires.

### Immunofluorescent Staining

For *in vitro* analysis, cells were fixed using a solution of 4% paraformaldehyde plus 4% sucrose in PBS (pH 7.2) for 15 min at room temperature at specific time points after the onset of treatments (Villarreal et al., 2014, 2016; Rosciszewski et al., 2019). After 15 min of fixation, the cells were washed three times with PBS and kept at 4°C. Before antibody incubation, fixated cells were permeabilized with 0.1% Triton-X in PBS for 15 min, followed by 30 min of incubation with blocking solution (5% normal horse serum in PBS). The cells were then incubated overnight with specific primary antibodies diluted in blocking solution. In specific experiments, microglia was detected using Tomato Lectin (TL) –FITC conjugate (Sigma), which was incubated together with the antibodies at a dilution of 1/1,000. After removal of primary antibodies, the cells were washed three times with PBS, followed by 4 h of incubation with fluorescent secondary antibodies. The cells were then washed three more times in PBS, incubated for 5 min with DAPI in PBS, and washed three more times with PBS.

For *in vivo* histological analysis, 4 days post-lesion (DPL), the animals were deeply anesthetized with ketamine/xylazine (90/10 mg/kg, i.p.) and perfused through the left ventricle as described (Aviles-Reyes et al., 2010). Brains were cryoprotected and snap-frozen, and coronal brain sections with a thickness of 25 μm were obtained using a cryostat (Leica). Free-floating sections were kept in a cryoprotective solution (30% glycerol and 20% ethylene glycol in 0.05 M phosphate buffer) at −20°C until use and processed in the free-floating state as previously described (Aviles-Reyes et al., 2010; Angelo et al., 2014). Primary and secondary antibodies were diluted in a solution with 3% normal horse serum and 0.3% Triton X-100 in PBS. Isotypic specific secondary antibodies labeled with Alexa 488 or 594 were used, and nuclear counterstaining was carried out with DAPI (0.1 μg/ml).

### Microscopy and Image Analysis and Processing

Imaging was conducted using an Olympus IX-81 microscope equipped with a DP71 camera (Olympus, Japan). *In vitro*, from each well, several images with a primary magnification of 20x were obtained and analyzed by a different person blinded to the treatment. All treatments and biological replicates of single experiments were imaged and documented in the same microscopy session. *In vivo*, for each animal, we obtained at least two images from the contralateral (CONTRA) and lesioned (ipsilateral, IPSI) hemispheres at a primary magnification of 20x and analyzed around 300 nuclei per image. Two 20x images cover most of the lesion caused by the cortical injury. Astrocytes and microglia were identified by their GFAP^+^ immunostaining, while microglia was identified by the Iba-1^+^ immunoreactivity.

Morphological analysis and cell counting (percentages of cells that are positive for a specific marker or protein) were conducted using Fiji (NIH, Schindelin et al., 2012). Images were merged, and cell counting was done using the Cell Counter plugin with channels Red, Green, and Blue overlapped.

Similarly, analysis of mean fluorescence intensity (MFI) for histone marks (typically, the red channel) was done in Fiji (Schindelin et al., 2012) with all channels overlapped to identify cell types. For each histone mark in astrocytes, a first region of interest (ROI) was delimited inside the DAPI-stained nucleus (nROI) of a GFAP-positive cell and a second one was delimited outside (oROI) to detect background signals. The mean fluorescence intensity of each single nucleus was calculated as nROI-oROI. In this way, we extracted a specific background signal in each analyzed nucleus. All nuclear measurements (>100 nuclei/image) from one image were averaged, and then, the means of at least three images were averaged to obtain the mean of a biological replicate. *In vivo*, the levels of histone modification were measured as they were *in vitro*, with the difference that *in vivo*, we also measured all the nuclei in a field independently of GFAP expression (Total). Since a specific histone modification could be changing in all cell types, we used this parameter to calculate a clean relative ratio of change in astrocytes due to the ischemic injury: Astrocyte/Total = [Astrocyte histone modification in ipsilateral (IPSI)/Total histone modification in ipsilateral (IPS)]/[Astrocyte histone modification in contralateral (CONTRA)/Total histone modification in CONTRA].

To estimate NF-κB activation, the percentage of a specific cell type with nuclear p65 (p65^nuc^) was calculated as the number of specific cell types (GFAP or Iba-1) with p65^nuc^ divided by the total number of specific cell types and multiplied by 100. The percentage of specific astrocytic or microglial morphological subtypes was calculated as the number of cells of a specific subtype divided by the total number of cells (GFAP or Iba-1) and multiplied by 100. The percentage of astrocytes and microglia was calculated as the number of cells immunoreactive for GFAP or Iba-1 divided by the total nuclei (DAPI) and multiplied by 100.

For figure preparation, images were processed (including size bar and labeling) using Fiji (Schindelin et al., 2012) and Inkscape (https://inkscape.org/). Bar size is indicated in each figure.

### Semiquantitative PCR

Cells plated and treated in 10-cm-diameter plates were collected using a fenol–chloroform base extraction protocol, with Prep-Zol (Inbio Highway, Tandil, Argentina) as the reagent and following the manufacturer’s indications. Total mRNA was converted into cDNA using a Genbiotech MMLV RT kit with oligo dTs (Genbiotech). PCR assays were performed using specific primers (Invitrogen): lL1β F: GAA​AGA​CGG​CAC​ACC​CAC​C, R: AAA​CCG​CTT​TTC​CAT​CTT​CTT​CT; C3 F: AGC​TTC​AGG​GTC​CCA​GCT​AC, R: GCT​GGA​ATC​TTG​ATG​GAG​ACG​C; actin F: CAC​CAC​TTT​CTA​CAA​TGA​GC, R: CGG​TCA​GGA​TCT​TCA​TGA​GG; and TBP F: ACCGTGAAT CTTGGCTGTA, R: CCG​TGG​CTC​TCT​TAT​TCT​CA. PCR amplicons were run in a 1.5% agarose gel using TAE buffer and imaged using a VersaDoc 4,000 imaging system (Bio-Rad, United States ). Each experiment included negative controls in which PCR reactions were performed in the absence of reverse transcriptase (Villarreal et al., 2014; Rosciszewski et al., 2017). Detailed PCR protocols are available from the authors under request.

Images were inverted and analyzed by NIH Fiji analysis for gels, where the optic density (OD) for each band is calculated. The value obtained for every band is first normalized to its housekeeping gene (actin or TBP). Then, the normalized value corresponding to the LPS treatment of each biological replicate was normalized to its control (untreated): LPS–Ctrl. Using one-sample t-test, we compared each normalized value to 0 (Ctrl=0).

### Statistical Analysis

While conducting glial primary culture, individual animals were processed separately, and cultures coming from different animals were considered different biological replicates (n). Values are plotted as mean and SEM.

Typically, cultures obtained from one animal were split into different wells or plates to conduct different treatments. The “n” number used in each experiment, which is specified in each figure legend, was never below three, and most experiments were repeated at least twice. In some experiments such as fluorescence measurements, the means from each condition were normalized to the control in order to obtain ratios (mean treated/mean control). In such cases, the control takes a value of one. In other experiments, such as PCR approaches and nuclear p65 analysis, the mean from the control conditions was subtracted from the treatment means to obtain the difference as a magnitude of change (mean treated–mean control). In such cases, the control takes a value of 0. In any case, each condition was compared to the untreated control using one-sample *t*-test against the hypothetical mean of one or 0, depending on the normalization. To compare between two conditions, a two-tailed Student’s t-test was conducted. For comparing non-normalized percentages, we used the nonparametric Kruskal–Wallis test. In all cases, a *p* value below 0.05 (*p* < 0.05) was considered as the parameter of significance. Statistical analysis and graph plotting were conducted using Prism GraphPad software.

## Results

### Phenotypic Conversion of Astrocytes and Microglia in Response to LPS Exposure

**FIGURE 1:**
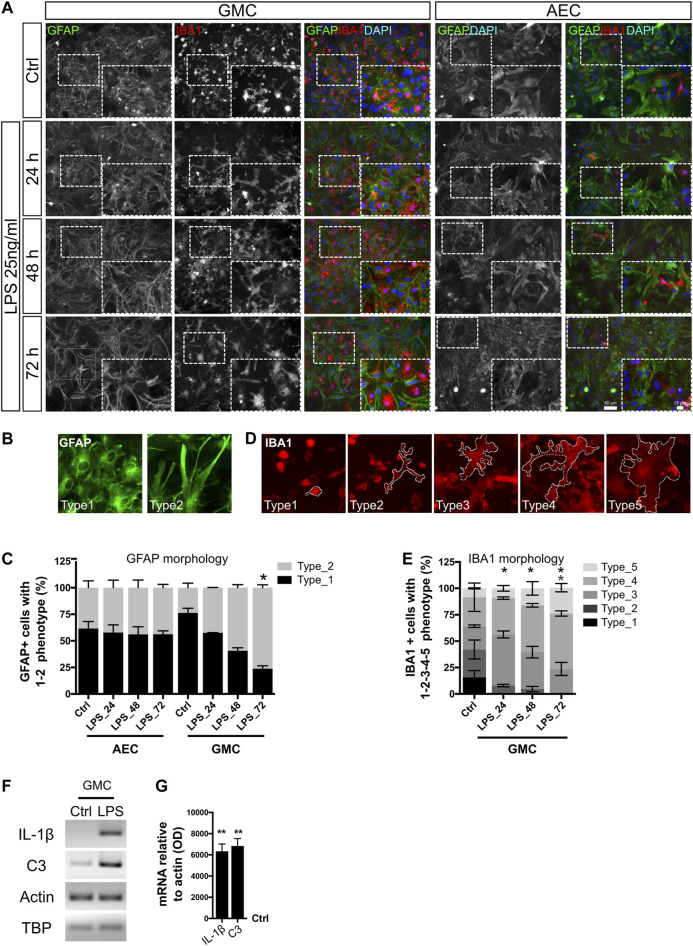
Phenotypic conversion of astrocytes and microglia in response to LPS. **(A)** Astrocyte-enriched cultures (AECs) and glial mixed cultures (GMCs) were incubated with LPS during the indicated time points in reduced-serum medium. To address cell morphology in response to LPS, we performed double immunofluorescence staining for GFAP and Iba-1 to distinguish astrocytes and microglia, respectively. **(B)** Representative images of the morphological classification of GFAP^+^ astrocytes *in vitro*. Type 1 astrocytes are polygonal-shaped astrocytes with few projections and type 2 astrocytes present highly stellated and elongated morphology. **(C)** Abundance of GFAP^+^ astroglial cell types for AEC or GMC at the analyzed time points. Bars show the mean percentage of three to four biological replicates with the standard error of the mean (SEM). Each time point was compared to the respective control (no LPS) using the nonparametric Kruskal–Wallis test with the statistical significance represented as *p < 0.05; *n* = 3. **(D)** Representative images of the morphological classification of Iba-1^+^ microgliocytes in five cell types, going from small bright and roundish microglial cells (type 1) to filamentous morphology (types 2–4) and ameboid flattened morphology (type 5) after LPS exposure. Dashed lines represent the cytoplasm limits of each microglial phenotype. **(E)** Distribution of microglial phenotypes in GMC exposed to LPS. Bars show the mean percentage of three biological replicates with the standard error of the mean (SEM). Each time point was compared to the respective control (no LPS) using the nonparametric Kruskal–Wallis test with the statistical significance represented as *p < 0.05; *n* = 3. **(F)** Representative bands for the indicated mRNAs are shown from GMC exposed for 20 h to LPS. Actin and TBP were used as housekeeping genes. **(G)** Graph showing relative changes of mRNAs after normalization to actin. LPS condition was compared to its control (no LPS) using one-sample *t*-test with the statistical significance represented as **p < 0.01, *n* = 4.

We initially addressed the morphological changes of astrocytes and microglia at different time points after LPS stimulus (24, 48, and 72 h) (Figure 1A). Astrocytes in GMC respond to LPS with phenotypic changes toward a more stretched and filamentous morphology. Figure 1A shows the typical morphological changes that were initiated after 24 h of LPS exposure but became statistically significant after 48 and 72 h. We conducted the experiments in glial mixed culture (GMC) containing 20% microglia and in astroglia-enriched cultures (AECs) containing less than 1% microglia. For quantitative studies, GFAP^+^ astrocytes were classified into two morphological phenotypes: the type 1 astrocytes were polygonal-shaped with few evident projections; while the type 2 astrocytes were highly stellated and elongated astrocytes (Figure 1B). Figure 1C and Supplementary Figure S1A show the increased abundance of the type 2 phenotype at different time points after LPS stimulus in GMC but not in microglia-depleted AEC.

In GMC cultures, microglia also responded to LPS with morphological changes, as evidenced by an increased proportion of ameboid (Caldeira et al., 2014) and large-size cells. To quantify the changes in the microglial phenotype, a morphological classification of five phenotypes was used (Figure 1D). Small bright and roundish microglial cells were observed under control conditions, and a 24 h LPS exposure induced rapid cytoplasmic remodeling of rounded microglia to adopt a highly filamentous phenotype (Figures 1A,E and Supplementary Figure S1B). Notably, LPS did not induce a statistically significant change in the total cell number, nor in the proportion between astrocytes, microglia, and the total cell number in GMC or AEC (Supplementary Figures S1C–E).

In all cases for AEC, the abundance of microglial cells was below 1%. Most imaged fields did not show any microglial cells, and some others showed very low counts (up to four cells). No evident phenotypic changes were observed in the very few Iba-1^+^ cells that remained in AEC, even under LPS exposure. However, microglial morphologies did not enter the classification used in GMC, showing already different basal morphological control conditions. These findings might suggest not only a microglial to astroglial interaction as described by Liddelow et al. (2017) but also a bidirectional communication between astrocytes and microglia to achieve morphological changes in response to LPS, probably through the release of soluble mediators such as C3, as already suggested (Lian et al., 2016).

We conclude that the astroglial phenotype changes dramatically in response to LPS only in the presence of microglia. Such changes start 24 h after exposure and gradually increase until 72 h after exposure. We then asked whether such a morphological phenotype correlates with the expression of genes related to the pro-inflammatory gain of function. mRNA analysis of pro-inflammatory genes showed an increase in the expression of IL-1β and C3, both markers of reactive and pro-inflammatory astroglia (Figures 1F,G). Taken together, astrocytes from GMC become reactive in response to LPS in the presence of microglia, showing features of a pathological remodeling with pro-inflammatory gain of function. The activation of C3 expression is not surprising in light of the finding that the C3 promoter has putative NF-κB binding sites (Lian et al., 2015). Furthermore, astrocytic C3 released to the extracellular space signals to neurons and microglia, mediating death and activation, respectively (Lian et al., 2015; Lian et al., 2016). In agreement, we have already reported that LPS (25 ng/ml)-treated GMC becomes neurotoxic to primary cortical neurons (Rosciszewski et al., 2019). Surprisingly, astrocytes in AEC showed a minor morphological response to LPS but a prominent increase in IL-1β expression, as assessed by RT-PCR. These results reinforce the idea that only a minor proportion of microgliocytes (less than 1%) are enough to provide the TLR4 receptor to subsequently induce the activation of neighboring astrocytes in the culture.

### Long-Lasting NF-κB Activation in Astrocytes

It has been previously shown by us and others that the NF-κB pathway mediates astrocytic pro-inflammatory gain of function (Lian et al., 2015; Rosciszewski et al., 2019). However, NF-κB activation is usually studied only during a short time period after stimulation. We next explored in detail the profile of NF-κB pathway activation in astrocytes and microglia following LPS exposure.

**FIGURE 2:**
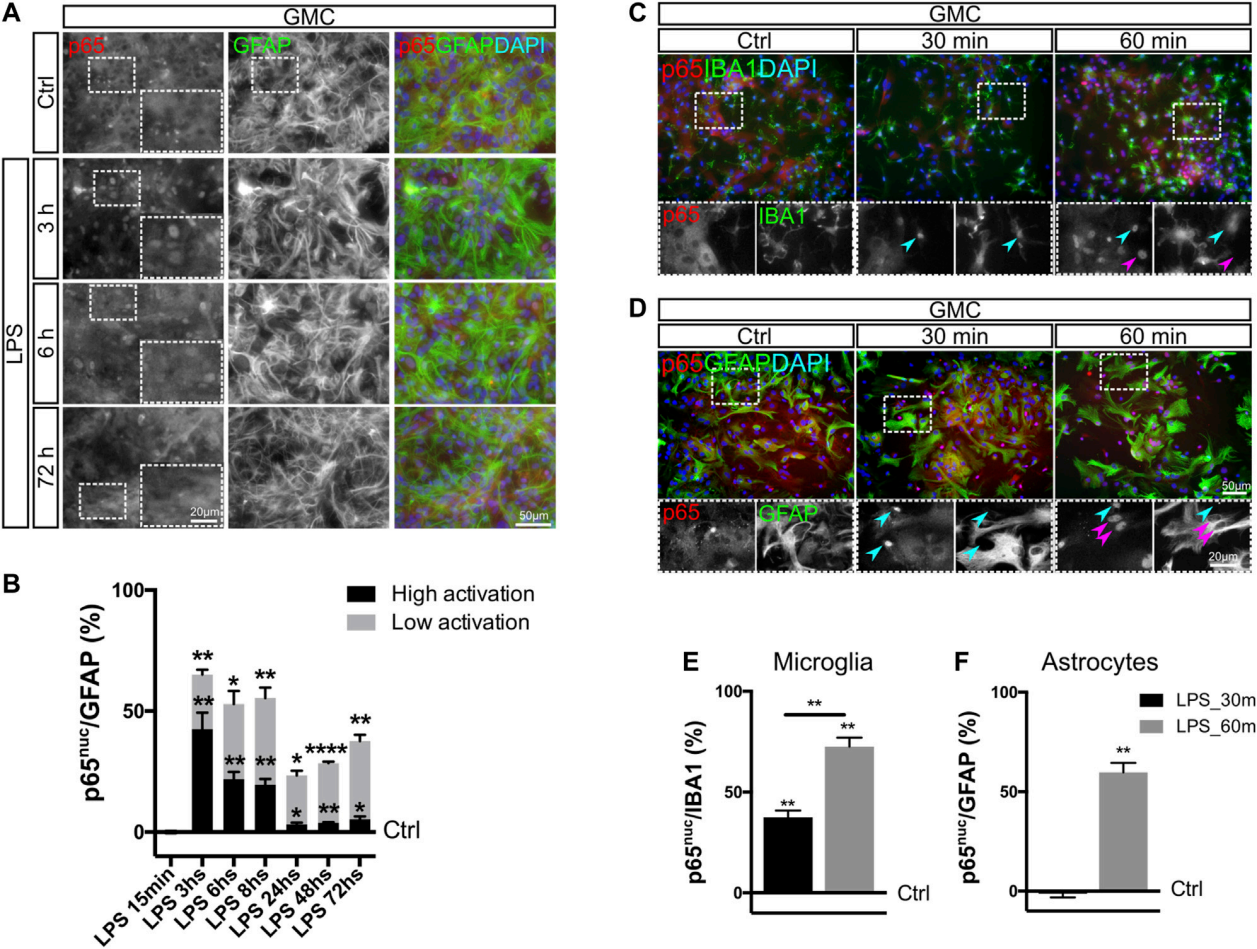
NF-κB activation in microglia occurs faster than in astrocytes. **(A)** Glial mixed cultures (GMCs) were incubated with LPS during the indicated time points in reduced-serum medium. To analyze NF-κB activation in astrocytes in response to LPS, we performed double immunofluorescence staining for GFAP and p65, determining the nuclear p65 localization (p65^nuc^). **(B)** Graph showing the proportion of GFAP^+^/p65^nuc^ cells from the total GFAP-positive cells after subtracting the percentage of GFAP^+^/p65^nuc^ under control conditions (Ctrl = 0). Bars show the mean percentage of three to four biological replicates with the SEM. Low activation corresponds to GFAP^+^ astrocytes, in which p65^nuc^ and cytoplasmic p65 (p65^cyt^) staining was homogeneous. High activation corresponds to GFAP^+^ astrocytes with distinguishable p65^nuc^ staining. Each time point was compared to the respective control (no LPS) using one-sample *t*-test with the statistical significance represented as *p < 0.05, **p < 0.01, or ****p < 0.0001; *n* = 3–4. **(C, D)** Glial mixed cultures (GMCs) were incubated with LPS during the indicated time points in reduced-serum medium. To address p65^nuc^ in microglia and astrocytes in response to LPS, we performed double immunofluorescence staining for Iba-1 and p65 **(C)** or GFAP and p65 **(D),** respectively. **(E)** Graph showing the proportion of Iba-1^+^/p65^nuc^–positive cells from the total Iba-1–positive cells after subtracting the percentage of Iba-1^+^/p65^nuc^ under control conditions (Ctrl = 0). Each time point was compared to the respective control (no LPS) using one-sample *t*-test with the statistical significance represented as **p < 0.01; n = 3. Comparison between treatments (time points) was conducted using Student’s t-test with the statistical significance represented as **p < 0.01; *n* = 3. **(F)** Graph showing the percentage of GFAP^+^/p65^nuc^-positive cells from the total GFAP-positive cells after subtracting the percentage of GFAP^+^/p65^nuc^ from the control (Ctrl=0). Each time point was compared to the respective control (no LPS) using one-sample *t*-test with the statistical significance represented as **p < 0.01; *n* = 3.

Keeping in mind that astrocytic phenotypic changes are still visualized after 72 h of LPS exposure, we then asked whether NF-κB was still active at that time point. For that purpose, we assessed the nuclear localization of the p65 subunit as a parameter of NF-κB activation (Villarreal et al., 2014) in GMC exposed to LPS. Using a reduced-serum medium allowed us to initiate these experiments with a low basal NF-κB activity, with less than 0.5% of astrocytes showing nuclear p65 (p65^nuc^) under control conditions and most cells having a clear negative nuclear staining (Figures 2A,D).

When analyzing cells with active NF-κB, we defined two levels of staining: *high activation*, corresponding to clear nuclear staining, and *low activation*, corresponding to homogeneous nuclear and cytoplasmic staining (non-negative nuclei) (Supplementary Figure S2B). Independently of the level of activation, graphs also indicate the total percentage of astrocytes with p65^nuc^ staining. The analysis of the time-course experiments showed that nuclear staining in astrocytes remains negative for 30 min (Supplementary Figure S2A) and becomes positive at 1 h of LPS exposure (Figures 2A,B). Specifically, we have observed that NF-κB in astrocytes shows an initial pattern of high activation (3–8 h), which is then replaced by a pattern of low but persistent activation at 24, 48, and 72 h (Figure 2B). We conclude that LPS exposure induces a long-lasting NF-κB activation in astrocytes when a significant amount of microglial cells is present in the culture.

### NF-κB Activation in Microglia Precedes NF-κB Activation in Astrocytes

Liddelow and colleagues (2017) demonstrated in a model of *in vivo* neonatal LPS injection that astrocyte conversion to the neurotoxic phenotype, which includes C3 expression, is microglia-dependent, suggesting that microglia are first responders to the pro-inflammatory stimulus. We explored if this response includes a faster NF-κB activation in microglia by following the dynamics of NF-κB activation in astrocytes and microglia from GMC. Using double immunofluorescent staining for Iba-1/p65^nuc^ and GFAP/p65^nuc^ at 30 and 60 min after LPS stimulus in microglia and astrocytes, respectively, we observed that microglia were fast responders to LPS, with p65^nuc^ staining significantly increasing already after 30 min of LPS exposure, while astrocytes required at least 60 min to reach a similar response (Figures 2C–F).

We concluded that NF-κB pathway activation after LPS exposure in mixed glial culture occurs sequentially, initially in microglia and then in astrocytes. This result is in line with previous findings showing that LPS-induced astroglial pro-inflammatory gain of function depends on the initial TLR4-mediated microglia activation (Liddelow et al., 2017). Taken together, our results indicate that the initial activation of microglia includes NF-κB activation and might represent a key step in crosstalk initiation following a pro-inflammatory stimulus.

### Microglia Abundance Determines Initial NF-κB Activation in Astrocytes

**FIGURE 3:**
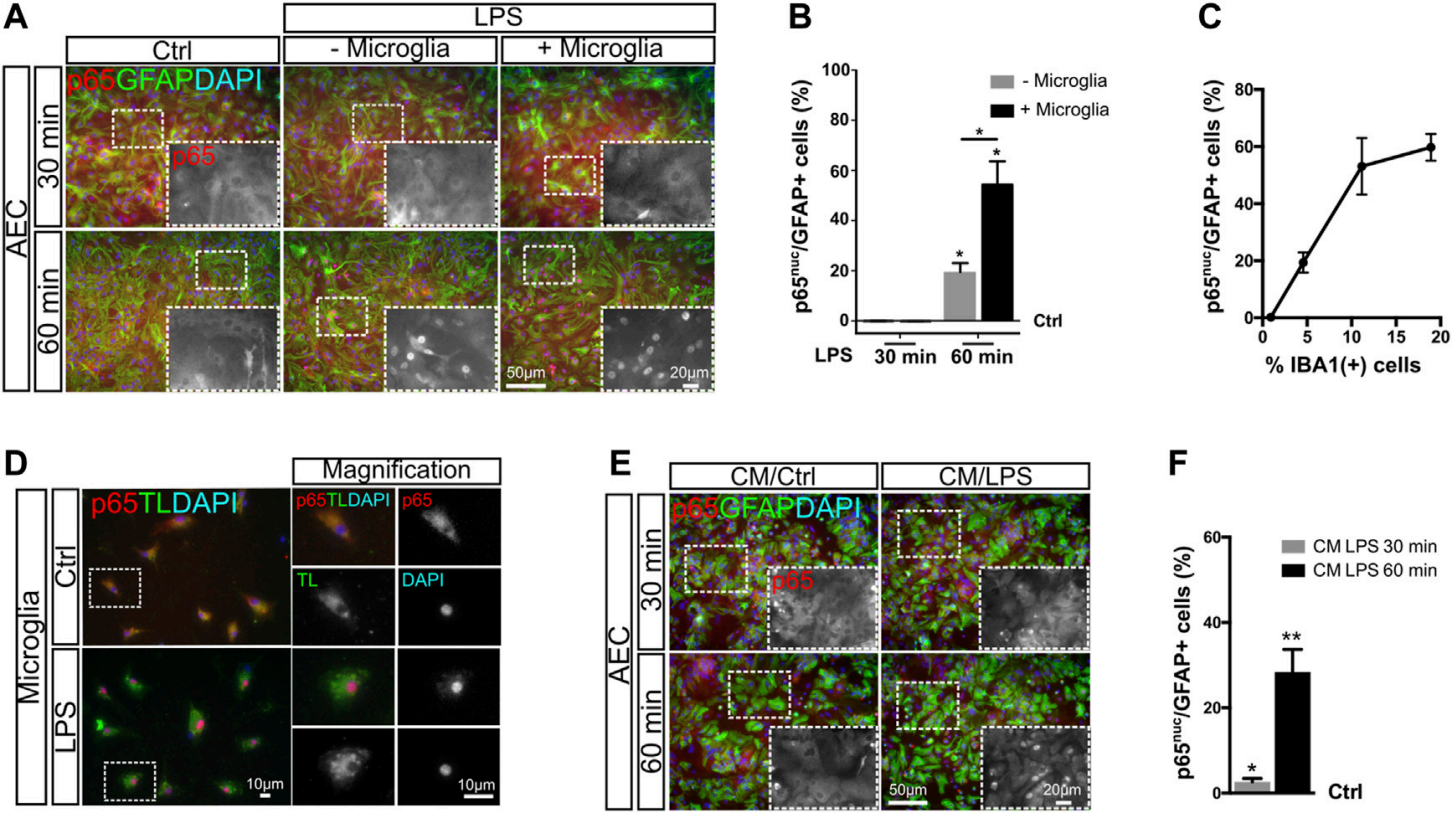
NF-κB initial activation in astrocytes depends on microglial soluble factors. **(A)** Astrocyte-enriched cultures (AECs) or microglia-reconstituted AEC (+microglia) were incubated with LPS during the indicated time points in reduced-serum medium. To analyze NF-κB activation in astrocytes in response to LPS, we performed double immunofluorescence staining for GFAP and p65, determining the nuclear p65 localization (p65^nuc^). **(B)** Graph showing the percentage of GFAP^+^/p65^nuc^-positive cells from the total GFAP-positive cells after subtracting the percentage of GFAP^+^/p65^nuc^ from the control (Ctrl = 0). Each time point was compared to the respective control (no LPS) using one-sample *t*-test with the statistical significance represented as *p < 0.05; *n* = 3. Comparisons between treatments were conducted using Student’s t-test with the statistical significance represented as *p < 0.05; *n* = 3. **(C)** Graph showing the proportion of GFAP^+^/p65^nuc^-positive cells plotted against the abundance of Iba-1^+^ cells from different experiments. Each dot corresponds to a different experiment and represents the mean of 3 different cultures. **(D)** Isolated microglia was incubated for 1 h with LPS in reduced-serum medium to obtain microglia-conditioned medium. To analyze NF-κB activation in isolated microglia in response to LPS, we performed double staining for FITC–tomato lectin and p65, determining the nuclear p65 localization (p65^nuc^). **(E)** Astrocyte-enriched cultures (AECs) with 1 h LPS-stimulated microglia-conditioned medium during the indicated time points. To analyze NF-κB activation in astrocytes in response to LPS, we performed double immunofluorescence staining for GFAP and p65, determining the nuclear p65 localization (p65^nuc^). **(F)** Graph showing the percentage of GFAP^+^/p65^nuc^-positive cells from the total GFAP-positive cells in CM-exposed astrocytes after subtracting the percentage of GFAP^+^/p65^nuc^ from the control (Ctrl=0). Each time point was compared to the respective control (no LPS) using one-sample *t*-test with the statistical significance represented as *p < 0.05, **p < 0.01; *n* = 6.

We noticed in our experimental model using AEC that LPS-induced NF-κB activation in astrocytes at 1 h is lower than that for astrocytes in GMC. This may suggest that at least for this initial time point, p65 nuclear translocation depends on the presence of microglia. We then asked whether the increasing microglia abundance in AEC cultures could modulate NF-κB activation. With this aim, we reconstituted AEC cultures by adding primary microglia, achieving an increase in the mean abundance of Iba-1 positive cells in 5% steps (going from 5 to 10%) (Supplementary Figures S3A,B). Using this paradigm, we observed that this 5% increase in microglia abundance promoted a 40% increase in the amount of astrocytes showing active NF-κB at 60 min after LPS exposure, as assessed by p65^nuc^ staining (Figures 3A,B). It is interesting to note that the addition of microglia did not accelerate the astroglial response to LPS at 30 min but increased the amount of astrocytes showing a significant NF-κB activation at 60 min (Figure 3B). Detailed images of these reconstituted glial cultures showed an initial NF-κB activation in astrocytes acquiring a *cluster-like* staining (Supplementary Figure S3C).

We compiled data from four different experiments and plotted the ratio of astrocytes with nuclear p65 staining (GFAP^+^/p65^nuc^) 60 min after LPS exposure vs. abundance of Iba-1^+^ microglia in culture and observed a clear positive correlation between both variables. As shown in Figure 3C, when the microglia abundance is lower than 1%, the proportion of GFAP^+^/p65^nuc^ cells is close to 0.5%. Increasing the microglial abundance to 5% microglia leads to a mean of 20% GFAP^+^/p65^nuc^ and 10% leads to above 50% of GFAP^+^/p65^nuc^ astrocytes. The 20% microglia corresponds to the abundance in GMC, and this level increases NF-κB activation up to a mean of 60% GFAP^+^/p65^nuc^ (Figure 3C).

We conclude that the abundance of microglia in the culture conditions the strength of the initial NF-κB activation in astrocytes, but it does not affect the speed of the initial NF-κB activation. It is worth noticing that *in vivo*, the number of microglial cells may remain constant for a short period of time, but then, microglial clone expansion may, in turn, affect astroglial reactivity.

### NF-κB Initial Activation in Astrocytes Depends on Microglial Soluble Factors

In GMC, microglia and astrocytes are in direct physical contact. We next address whether physical contact between microglia and astrocytes was necessary to trigger the initial p65 nuclear translocation induced by LPS exposure. With this aim, we exposed purified microglia to LPS for 60 min and collected the conditioned medium. Microglia activation was confirmed by immunofluorescent double staining for Tomato Lectin/p65^nuc^ (Figure 3D). We then exposed microglia-depleted AEC to microglia-conditioned medium and analyzed the NF-κB activation by quantifying the GFAP^+^/p65^nuc^ cells after 30 and 60 min of incubation. We observed a significant increase in the GFAP^+^/p65^nuc^ cells at both time points, it being higher at 60 min for astrocytes exposed to microglia-conditioned medium (Figures 3E,F). Although at 30 min the response was very low (below 3% on average), it was consistent across the biological replicates, again showing this intriguing delay in NF-κB activation in astrocytes. It is important to notice that these AECs are depleted from microglia, and consequently, they do not respond to LPS in terms of nuclear p65 (Supplementary Figures S3D,E).

We concluded that the initial NF-κB activation is triggered by soluble factors released from the microglia and does not require physical contact between astrocytes and microglia.

### Chromatin Remodeling in Astrocytes is Microglia-Dependent

After engaging in cellular crosstalk with LPS-treated microglia, astrocytes change their phenotype toward a reactive and pro-inflammatory state. This was evidenced by morphological changes, activation of NF-κB, and expression of pro-inflammatory genes. Such a phenotype remains stable for at least 72 h after LPS exposure, a time point after the initial peak of NF-κB activation (Figures 1, 2). NF-κB is known to promote histone acetylation (Berghe et al., 1999; Mukherjee et al., 2013), and it is widely accepted that histone acetylation promotes an active state of the chromatin. Given that astrocyte phenotypic changes correlate with major changes in gene expression, we then asked whether this is reflected in global changes of histone acetylation. We also asked if those possible changes were, as are NF-κB activation and phenotype conversion, microglia-dependent.

**FIGURE 4:**
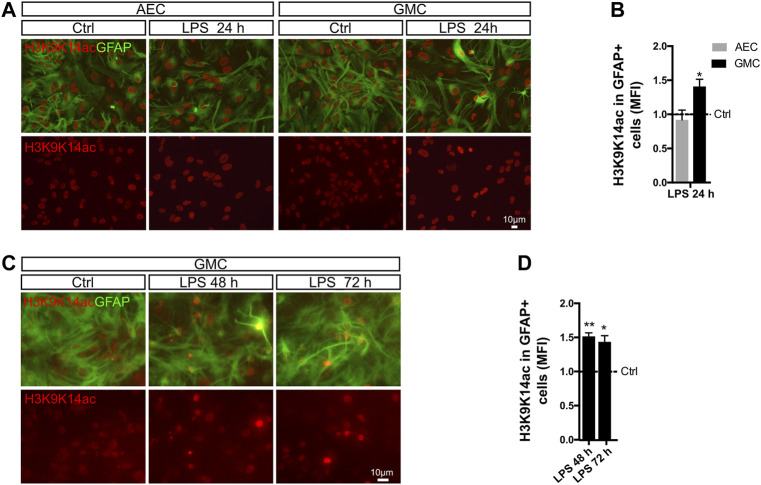
Histone acetylation in astrocytes is microglia-dependent. **(A)** Astrocyte-enriched cultures (AECs) or glial mixed cultures (GMCs) were incubated with LPS for 24 h in reduced-serum medium. To address changes in the global levels of histone 3 acetylation in astrocytes in response to LPS, we performed double immunofluorescence staining for GFAP and acetylated lysins 9 and 14 of histone 3 (H3K9K14ac). **(B)** Graph showing the mean fluorescence intensity (MFI) for H3K9K14ac in the nuclei of GFAP-positive cells after normalizing to the control (Ctrl = 1). Each time point was compared to the respective control (no LPS) using one-sample *t*-test with the statistical significance represented as *p < 0.05. *n* = 3–6. **(C)** Glial mixed cultures (GMCs) were incubated with LPS during the indicated time points in reduced-serum medium. To address changes in the global levels of histone 3 acetylation in astrocytes in response to LPS, we performed double immunofluorescence staining for GFAP and acetylated lysins 9 and 14 of histone 3 (H3K9K14ac). **(D)** Graph showing the mean fluorescence intensity (MFI) for H3K9K14ac in the nuclei of GFAP-positive cells after normalizing to the control (Ctrl = 1). Each time point was compared to the respective control (no LPS) using one-sample *t*-test with the statistical significance represented as *p < 0.05, **p < 0.01; *n* = 3.

With this aim, we treated AEC and GMC with LPS and monitored histone 3 acetylation (H3Ac) intensity in astrocytes by conducting double immunofluorescent staining for GFAP and H3K9K14ac. Notably, we observed a significant increase in H3K9K14ac intensity after 24 h in astrocytes only in the presence of microglia (Figures 4A,B). Furthermore, the increase in H3K9K14ac intensity in astrocytes remained for at least 72 h (Figures 4C,D).

It should be highlighted that the LPS-mediated increased H3K9K14ac fluorescence intensity appears to be highly heterogeneous within the astroglial population, with a subpopulation of astrocytes shifting toward an increase in H3K9K14ac intensity. Overall, our results indicate an increase in global histone acetylation in LPS-induced reactive astrocytes. Interestingly, H3K9K14ac has been previously associated with p65 occupancy at regulatory regions (Federman et al., 2013).

**FIGURE 5:**
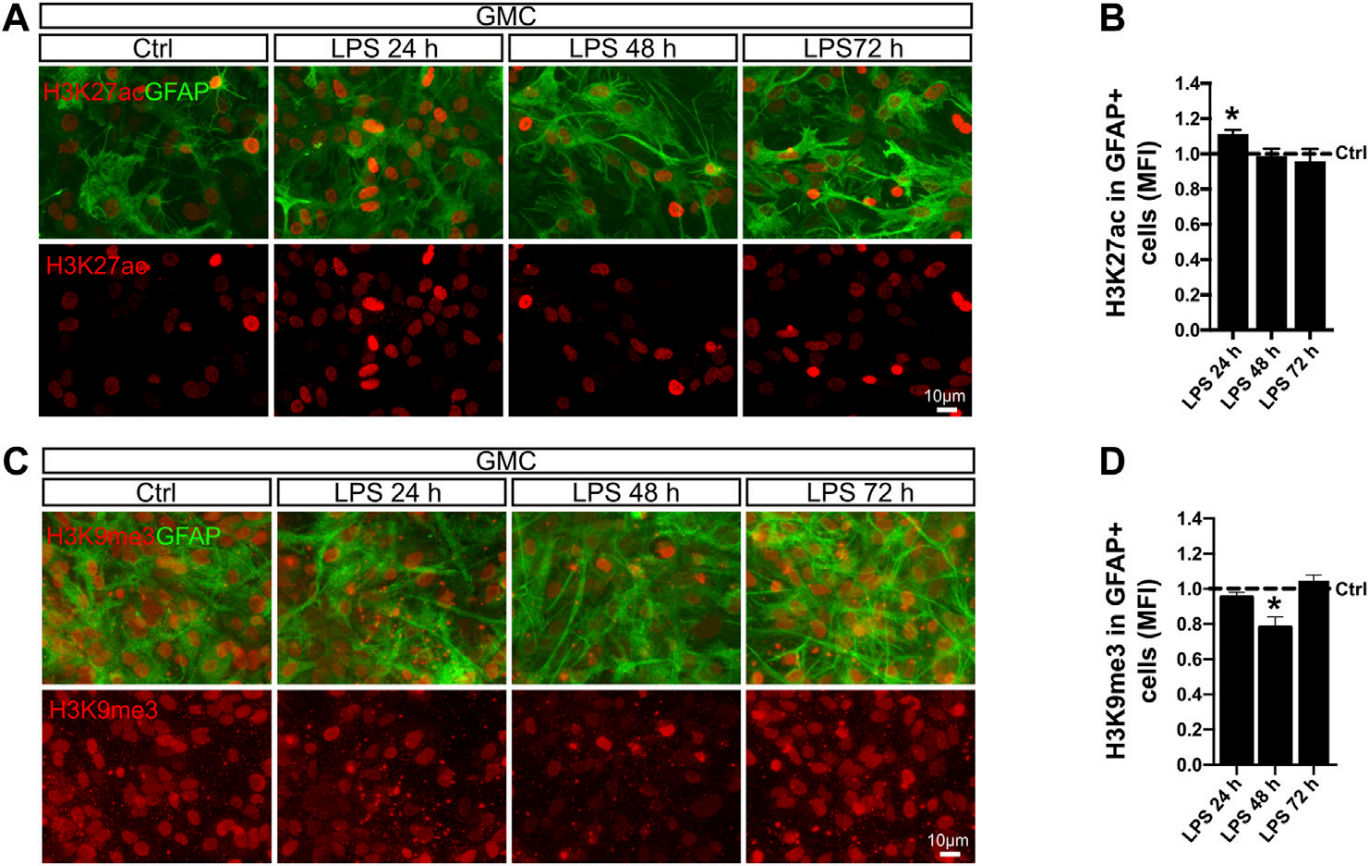
LPS promotes global chromatin remodeling in astrocytes in a time-dependent manner. **(A)** Glial mixed cultures (GMCs) were incubated with LPS during the indicated time points in reduced-serum medium. To address changes in histone 3 acetylation at lysin 27 (H3K27ac) in astrocytes in response to LPS, we performed double immunofluorescence staining for GFAP and H3K27ac. **(B)** Graph showing the mean fluorescence intensity (MFI) for H3K27ac in the nuclei of GFAP-positive cells after normalizing to the control (Ctrl = 1). Each time point was compared to the respective control (no LPS) using one-sample *t*-test with the statistical significance represented as *p < 0.05; *n* = 3. **(C)** Glial mixed cultures (GMCs) were incubated with LPS during the indicated time points in reduced-serum medium. To address changes in trimethylated histone 3 lysin 9 (H3K9me3), immunofluorescence staining for GFAP and H3K9me3 was performed. **(D)** Graph showing the mean fluorescence intensity (MFI) for H3K9K9me3 in the nuclei of GFAP-positive cells after normalizing to the control (Ctrl = 1). Each time point was compared to the respective control (no LPS) using one-sample t-test with the statistical significance represented as *p < 0.05, *n* = 4.

Next, we studied H3K27ac mark, which has been linked to p300 acetyltransferase activity (Weinert et al., 2018). This enzyme is a known interaction partner of the p65 NF-κB subunit in the regulation of pro-inflammatory genes (Berghe et al., 1999). We therefore analyzed the levels of H3K27ac in astrocyte nuclei after LPS exposure and observed that the global intensity of H3K27ac slightly increased after 24 h (Figures 5A,B). Increased transcriptional activity is usually accompanied by decreased repressive marks such as H3K9me3, a mark for heterochromatin (Becker et al., 2016). Using the same experimental approach, we addressed H3K9me3 intensity in astrocytes by conducting double immunofluorescent stainings for GFAP and H3K9me3. Interestingly, we observed a transient but significant decrease in the levels of H3K9me3 intensity after 48 h in astrocytes from GMC, which recovered control values by 72 h (Figures 5C,D).

We conclude that exposure to LPS triggers global long-term changes in H3Ac and temporal changes in H3K27ac and H3K9me3 in astrocytes when microglia is available. A global increase in histone acetylation together with dynamic changes of specific epigenetic marks might indicate actual chromatin reconfiguration in astrocytes that are engaged in a pro-inflammatory gain of function after LPS exposure.

### Brain Injury Promotes Chromatin Remodeling in Reactive Astrocytes

**FIGURE 6:**
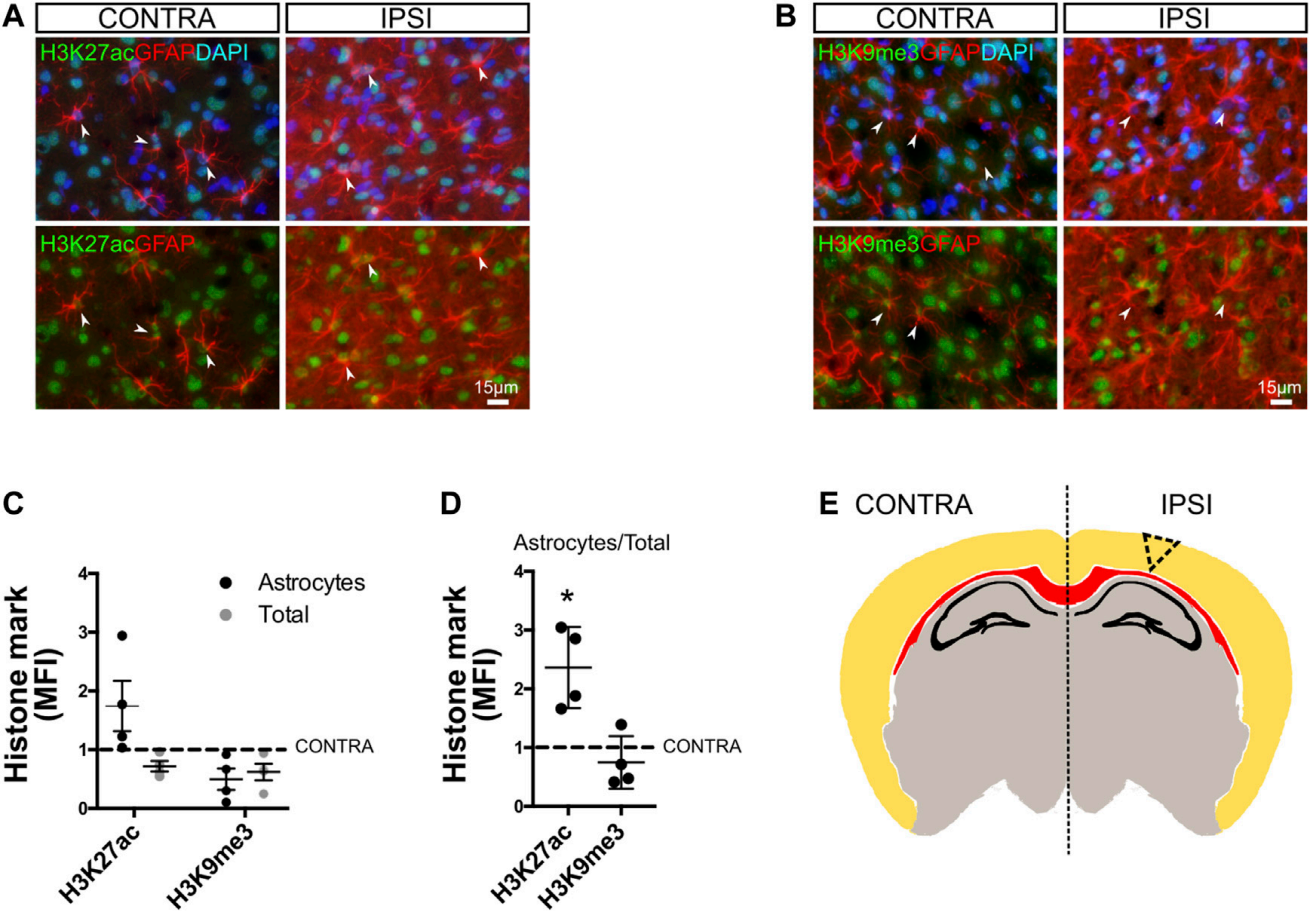
Chromatin remodeling in reactive astrocytes after cortical ischemic injury *in vivo*. **(A)** Brain sections from animals subjected to cortical devascularization were processed for immunofluorescence staining. To address changes in the global levels of H3K27ac in astrocytes in response to damage, we performed double immunofluorescence staining for GFAP and H3K27ac. Images show representative fields of the contralateral (CONTRA) hemisphere and the ipsilateral (IPSI) hemisphere with and without nuclear DAPI counterstaining. The arrowheads indicate representative astrocytes with visible nuclei. Here, GFAP intensity was enhanced using FIJI software to visualize cells, and intensity is not representative of the reactivity state. **(B)** Similar to A, we performed double immunofluorescence staining for GFAP and H3K9me3. **(C)** Graph showing the mean fluorescence intensity (MFI) for H3K27ac and H3K9me3 in the nuclei of GFAP-positive cells (black dots = astrocytes) or in other cell types nuclei (gray dots = total) after normalizing to the control (Ctrl = 1). **(D)** Graph showing the mean fluorescence intensity (MFI) for H3K27ac and H3K9me3 in the nuclei of GFAP-positive cells relative to the total nuclei and after normalizing to the control (Ctrl = 1). Average from the hemisphere of one animal was compared to the respective contralateral hemisphere (CONTRA) using one-sample t-test with the statistical significance represented as *p < 0.05, n = 4. **(E)** Schematic representation of a coronal section from a murine brain indicating the site of the cortical lesion (triangle dotted lines). The corpus callosum is shown in red and the hippocampal pyramidal cell layer and dentate gyrus are shown in black.

Long-lasting astroglial phenotypical remodeling is a hallmark of focal brain injury. Focal brain ischemia in rodents reproduces several features of human ischemic brains, with extended reactive gliosis and a prominent ischemic core surrounded by penumbra (Aviles-Reyes et al., 2010; Villarreal et al., 2011, 2016). Reactive astrogliosis rapidly occurs, and NF-κB activation has been evidenced in the tissue surrounding the ischemic core, giving rise to the pro-inflammatory gain of function in a subset of the astroglial population (Rosciszewski et al., 2017) that is notably stable even when cultured *ex vivo* (Villarreal et al., 2016). Based on these reports and the present findings, we explored if the chromatin remodeling events that we described *in vitro* for mixed glial cultures were also present *in vivo* in an experimental model of brain ischemia by cortical devascularization. For that purpose, we subjected animals to the pial disruption model that produces a cortical devascularizing lesion (Aviles-Reyes et al., 2010; Villarreal et al., 2011; Villarreal et al., 2016). As shown in Figure 6, we observed at 4 days post-lesion that there were an increased abundance of the epigenetic mark H3K27ac and a decrease in H3K9me3 mark, specifically in GFAP^+^ astrocytes from the ischemic penumbra, in all analyzed animals when compared to the uninjured hemisphere (Figures 6A–C). We also analyzed the histone modification intensity in all nuclei and found, for both marks, a slight decrease in abundance (Figure 6C). We then calculated the net amount of change in histone modification in GFAP^+^ cells by normalizing this value to total intensity. Results showed that H3K27ac in astrocytes was significantly increased, while H3K9me3 did not change (Figure 6D). This suggests that the H3K27ac increase is astrocyte-specific and is a penumbra-associated phenomenon *in vivo* (Figure 6E).

We conclude that chromatin remodeling events are a common feature that reactive astrocytes exhibit, both *in vitro* and *in vivo*.

## Discussion

Reactive astrogliosis is a generic response of astrocytes triggered by different types of CNS injuries. After decades of conflicting reports attributing beneficial or detrimental effects, results from the last decade obtained using transcriptomic approaches have shown that reactive gliosis is a complex phenomenon that involves astroglial remodeling and deleterious effects occurring when astrocytes lose their homeostatic features and/or suffer a pro-inflammatory gain of function (Zamanian et al., 2012; Anderson et al., 2016; Escartin et al., 2021). Once initiated by diffuse or focal injury, reactive astrogliosis becomes stable over time with glial scar formation (Sofroniew, 2009; Orr and Gensel, 2018) or *ex vivo* cultures of reactive astrocytes being exquisite examples of such stability (Díaz-Amarilla et al., 2011; Villarreal et al., 2016).

The prominent role of NF-κB in reactive astrogliosis and specifically in astroglial pro-inflammatory gain of function pointed us toward studying the potential chromatin remodeling effects downstream of NF-κB activation. Given the fact that large transcriptomic changes were reported for reactive astrocytes, we here explored, in a global manner, the levels of astrocytic acetylated histone 3 at lysins 9 and 14, which were already linked to NF-κB pathway activation (Federman et al., 2013). We found that astroglial H3K9K14ac levels are elevated after LPS exposure and remain increased for several days only in the presence of microglia. Similarly, H3K27ac showed an acute increase in astrocytes by 24 h after LPS exposure. These results are of significant interest to the current work, since H3K27ac mark is added by acetyltransferase p300, which is in turn a very well-known interaction partner of NF-κB in active regulatory regions (Weinert et al., 2018). It is likely that these activating marks allow the transcription of genes involved in reactive astrogliosis. Interestingly, our present *in vivo* results have shown that these pathways are also active in a model of brain ischemia by pial disruption.

NF-κB–dependent chromatin remodeling is associated with histone acetylation at active transcriptional sites. In addition, H3K9me3, a mark for heterochromatin and transcription silencing (Becker et al., 2016), resulted in temporarily decreased reactive astrocytes being remodeled to the pro-inflammatory gain of function by LPS exposure. Overall, we hypothesize that the increases in activating marks together with a decrease in repressive marks in the same regions allow stable chromatin reconfiguration in reactive astrocytes. Furthermore, homeostatic genes or genes that are not involved in reactive phenotypes are likely to be repressed. This means that while some regions become part of active transcription, others may become repressed. This may explain the fact that the observed changes (H3K27ac and H3K9me3) are temporal, when addressed globally. Future experiments will address such changes on a genome-wide basis at a single-gene resolution.

**FIGURE 7:**
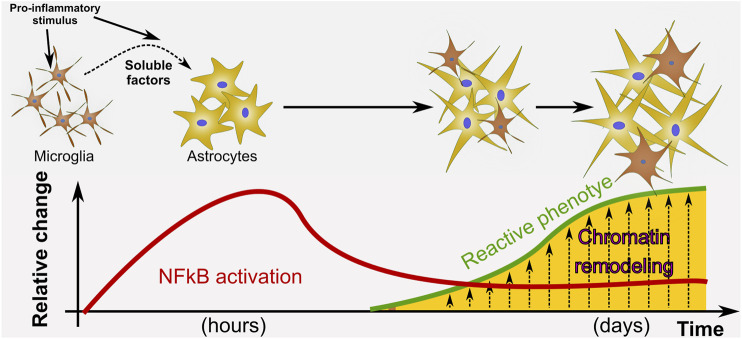
Proposed model of astrocyte phenotype conversion. We propose a model in which a pro-inflammatory stimulus such as LPS activates NF-κB in astrocytes and promotes pathological remodeling of reactive astrocytes with pro-inflammatory gain of function only when microglia is present. In our *in vitro* paradigm, cell-to-cell communication to activate NF-κB nuclear translocation does not depend on physical contact with microglia but on the release of soluble factors. We propose that after NF-κB initial activation, the astrocyte reactive phenotype is sustained by epigenetic changes at the chromatin level.

Interestingly, the presently reported findings, highly indicative of astroglial chromatin remodeling, are paralleled with the expression of pro-inflammatory genes that have been shown to be detrimental for neuronal survival (Rosciszewski et al., 2019). Chromatin remodeling is likely to be occurring in astrocytes during the process of pathological remodeling that leads to the pro-inflammatory gain of function induced by LPS exposure (Figure 7).

Our results also provide new evidence with regard to microglia–astrocyte crosstalk under inflammatory conditions induced by exposure to a powerful pro-inflammatory molecule such as bacterial LPS. First, we show that NF-κB activation in microglia precedes NF-κB activation in astrocytes. It has been stated that microglia mediates astroglial pro-inflammatory conversion, leading to the idea of temporal causality between the responses of both cell types (Liddelow et al., 2017). However, to the best of our knowledge, this was never shown in terms of NF-κB activation. Our present results provide an interesting insight in terms of the temporal activation of the NF-κB pathway in the different glial cell types. Obvious candidates for this initial fast microglial response are the pattern recognition receptors (PRRs) of the toll-like receptor family, specifically TLR4, which is the prototypical LPS receptor expressed in microglia. We further described that astrocyte initial NF-κB activation, and not just activation strength, is microglia-dependent. Direct contact between microglia and astrocytes might have a role in astrocyte initial activation; however, our results suggest that microglia signaling to astrocytes under these conditions is mediated by soluble factors, which is in agreement with previous results produced by Liddelow et al. (2017). As expected, the concentration threshold of soluble factors probably depends on the amount of microglia in culture; thus, the astroglial response is more intense when increasing the microglial cell number. Moreover, the fact that astrocytes respond to conditioned medium already at 30 min suggests that the required 60 min period in GMC is crucial for the release of soluble factors from the microglia. Taken together, our results indicate that astrocytes might not be able to initially respond to LPS in the absence of microglial soluble factors, but after the initial stimulation, cell-autonomous mechanisms prevail, and astrocytes follow the reactive gliosis gene expression program. The identity of the soluble factors released by microglia was partially revealed in previous works that determined that IL-1α, TNFα, and C1q are involved in microglia-dependent astrocytic activation in rodent glia or in human induced pluripotent stem cell–derived astrocytes (Liddelow et al., 2017; Santos et al., 2017). However, it still remains to be determined if these cytokines are able to activate the full downstream epigenetic remodeling presently described following the pro-inflammatory gain of function induced by LPS. Our group will address this topic in future studies.

Although most of the attention has been focused on microglial to astroglial communication, astrocytes are also able to signal to microglia, propagating the inflammatory response in time and space. Particularly, pro-inflammatory astrocytes show increased C3 expression, which is released to the extracellular space (Liddelow et al., 2017), C3 being another NF-κB–dependent gene and having microglia C3R on its membrane (Lian et al., 2015). In agreement, we found that LPS-exposed astrocytes induced C3 gene expression and that microglial cells from GMC but not from AEC showed profound morphological alterations following LPS exposure. This might be indicating that microglia also require astrocyte activation in order to become pro-inflammatory.

Histone acetylation is a promising pharmacological target to modulate gene transcription of cells in pathological contexts, and great advances have been made in the cancer research field, producing a large number of compounds that can be repurposed for developing new treatment strategies (Hontecillas-Prieto et al., 2020). The use of HDAC inhibitors has been generically proposed to modulate the neuroinflammatory response (Chuang et al., 2009; Durham et al., 2017; Yang et al., 2017; Neal and Richardson, 2018); however, even if HDAC inhibition results in a global anti-inflammatory effect, a more detailed analysis clearly depicting the effects in the different cell types of the neurovascular unit is still pending. Additionally, molecular targets are not always clearly defined for these drugs, since modifications of HDAC non-histone targets are also being modulated by these approaches (Glozak et al., 2005; Singh et al., 2010). Furthermore, commonly used HDAC inhibitors like VPA are also known to have several non-HDAC targets (Soria-Castro et al., 2019). This last issue has led to the design of novel and improved HDAC inhibitors (Yang et al., 2017; Ziemka-Nalecz et al., 2018).

In summary, our results show that NF-κB activation in microglia precedes NF-κB engagement in astrocytes when cells are exposed to LPS. Microglia abundance is relevant for initial NF-κB activation in astrocytes, and soluble factors seem to be essential for this crosstalk. Microglia is also required for promoting chromatin remodeling events in astrocytes that are concomitant with increased pro-inflammatory cytokine expression and neurotoxic effects, both features of the pathological remodeling with pro-inflammatory gain of function. *In vivo* experimental ischemia also shows the activation of this downstream NF-κB signaling pathway and chromatin remodeling events in reactive astrocytes from the ischemic penumbra. Taken together, these results strongly suggest that microglia is not only required for astrocyte initial activation and acquisition of a pro-inflammatory phenotype but also for sustaining astrocyte phenotypic pathological remodeling with pro-inflammatory gain of function through mechanisms of chromatin remodeling. The identification of this new potential pharmacological target to control pathological astroglial remodeling opens a new avenue for future therapeutic research in CNS diseases where the detrimental role of astrocytic pro-inflammatory conversion has been evidenced.

## Data Availability

The original contributions presented in the study are included in the article/Supplementary Material; further inquiries can be directed to the corresponding authors.

## Abbreviations

5-FU, 5-fluorouracyl; BSA, bovine serum albumin; CNS, central nervous system; DAMP, damage-associated molecular pattern; DMEM, Dulbecco’s modified Eagle medium; GFAP, glial fibrillary acidic protein; IL-1β, interleukin 1-beta; IL-1α, interleukin 1-alpha; Iba-1, ionized calcium binding adaptor molecule 1; NF-κB, nuclear factor kappa B; PRR, pattern recognition receptor; CM, conditioned medium; TLR4, toll like receptor 4; VPA, valproic acid; AEC, astrocyte-enriched culture; GMC, glial mixed culture; HATs, histone acetyltransferases; HDACs, histone deacetylases; TNFα, tumor necrosis factor alpha; C1q, complement component 1q; LPS, lipopolysaccharide; H3, histone 3; H3K9K14ac, acetylation of histone 3 at lysins 9 and 14 (activating mark); H3K27ac, acetylation of histone 3 at lysin 27 (activating mark); and H3K9me3, trimethylation of histone 3 at lysin 9 (repressive mark).
