# Supplementary figures and images for: Pathological Neuroinflammatory Conversion of Reactive Astrocytes Is Induced by Microglia and Involves Chromatin Remodeling
Source: Front Pharmacol. 2021 Jun 21;12:689346. doi: 10.3389/fphar.2021.689346 (PMC8255379; doi:10.3389/fphar.2021.689346)

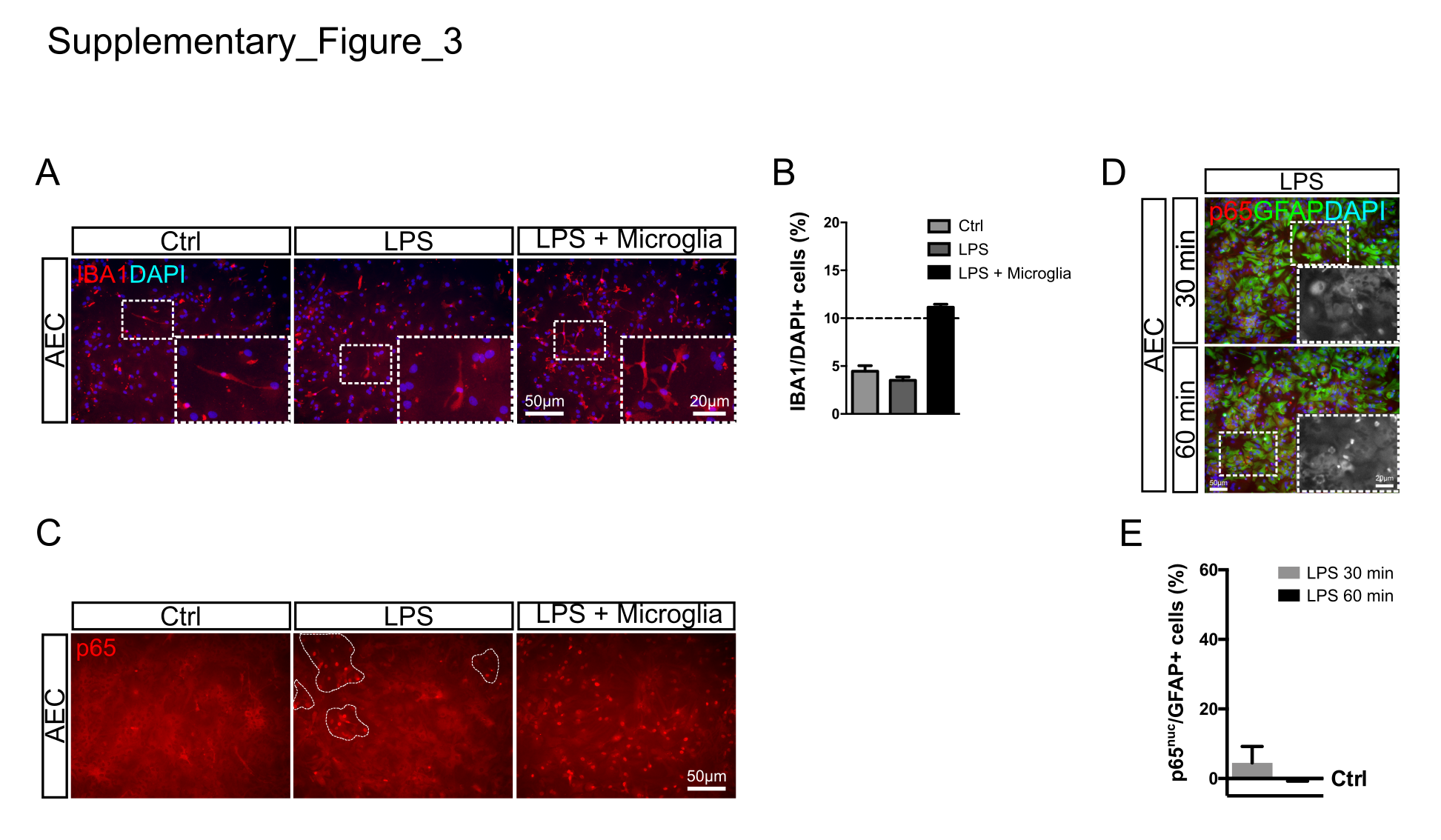

Supplement: Supplementary file 1 [file Image3.TIF]

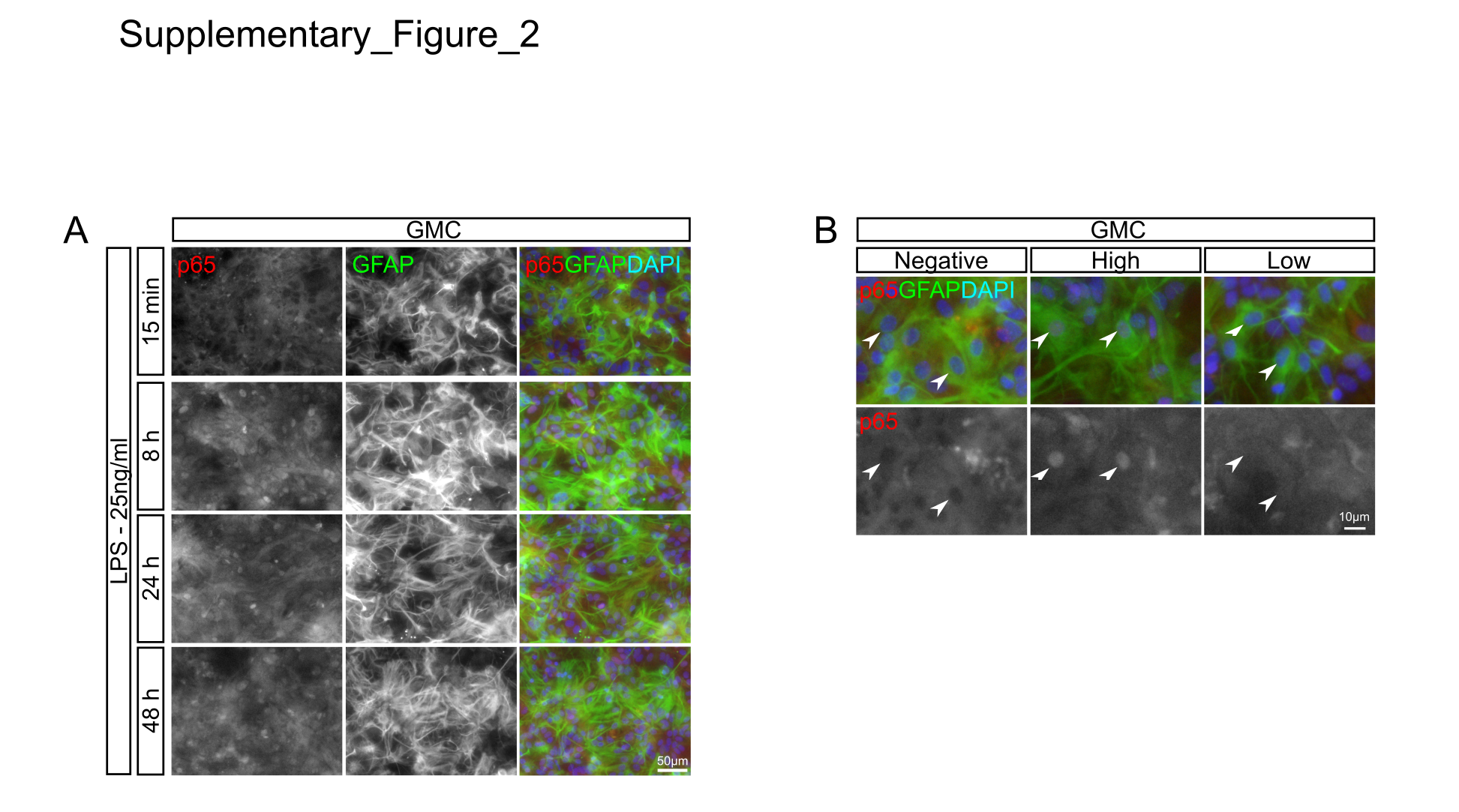

Supplement: Supplementary file 2 [file Image2.TIF]

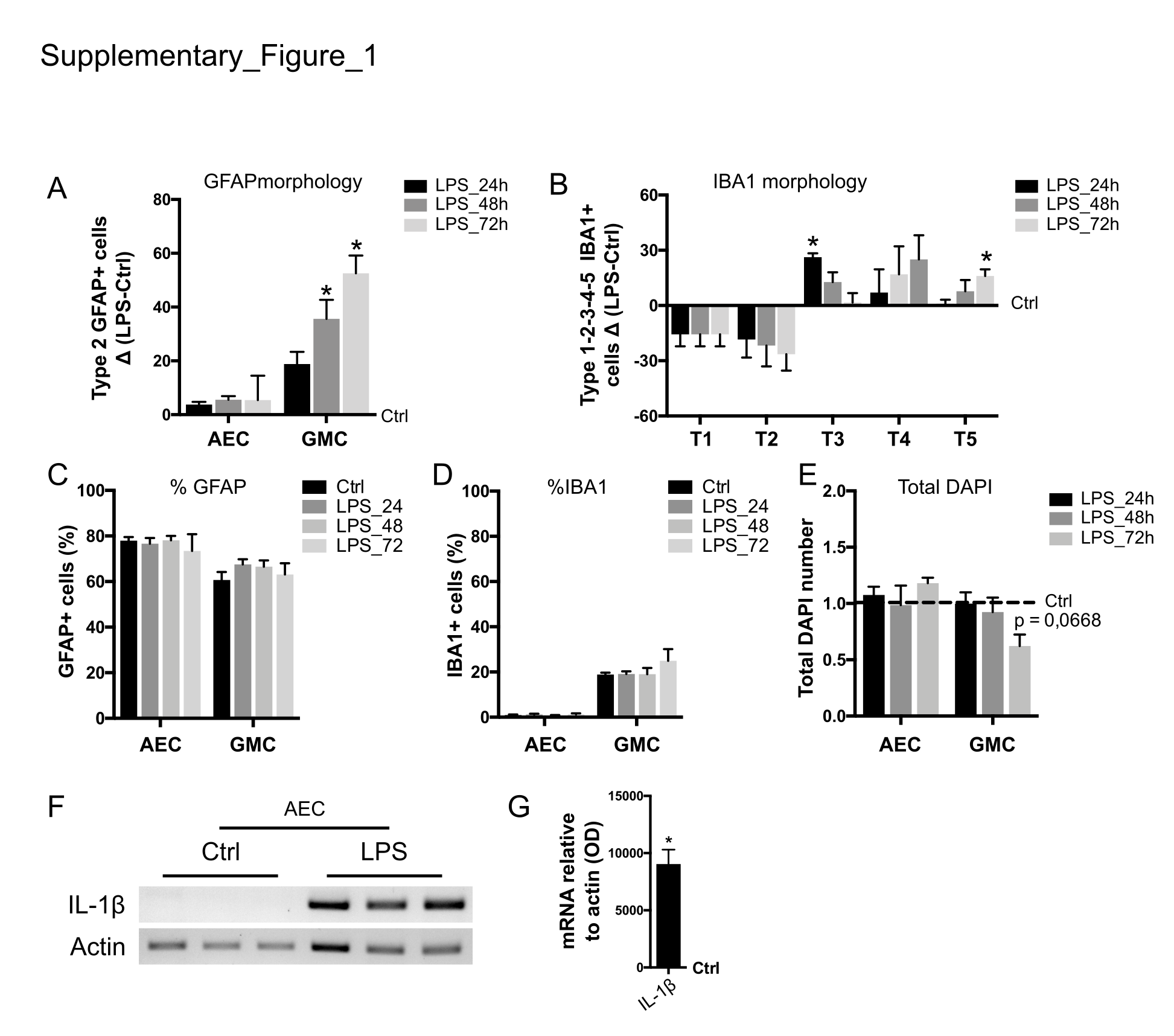

Supplement: Supplementary file 3 [file Image1.TIF]
