# Supplementary material for: Pathological Neuroinflammatory Conversion of Reactive Astrocytes Is Induced by Microglia and Involves Chromatin Remodeling
Source: Front Pharmacol. 2021 Jun 21;12:689346. doi: 10.3389/fphar.2021.689346 (PMC8255379; doi:10.3389/fphar.2021.689346)
Supplement: Supplementary file 4 [file DataSheet1.docx]

**Legends to Supplementary Figures**

**Supplementary Figure 1**

**A:**  Graph shows the increase in type 2 (reactive astrocyte) GFAP positive cells for each culture conditions (AEC and GMC) and for all analyzed time points. The increase was calculated by subtracting control values from each treatment (Ctrl=0). Each condition (time points) was compared to it's control (no LPS) using a One sample T-test with statistical significance represented according to p value: ^*^p < 0.05. n=3. **B:** Graph shows the increase in all types of IBA1 positive cells in GMC for all analyzed time points. The increase was calculated by subtracting control values from each treatment (Ctrl=0). Each condition (time points) was compared to its control (no LPS) using a One sample T-test with statistical significance represented according to p value: ^*^p < 0.05 n=3. **C:** Graphs shows percentage of GFAP positive cells for each culture conditions (AEC and GMC) and for all analyzed time points **D:** Graphs shows percentage of IBA1 positive cells for each culture conditions (AEC and GMC) and for all analyzed time points. **E:** Graphs shows total number of cells expressed as total nuclei (DAPI)/field for each culture conditions (AEC and GMC) and for all analyzed time points. **F:** Representative bands for the indicated mRNAs are shown from AEC exposed 20 h to LPS. Actin was used as housekeeping gene **G:** Graphs show relative changes of IL1B mRNA after normalization to actin. LPS condition was compared to its control (no LPS) using a One sample T-test with statistical significance represented according to p value: *p < 0.05. n=3.

**Supplementary Figure 2**

**A:** Glial mixed cultures were incubated with LPS during the indicated time points in reduced-serum medium. To address nuclear p65 in astrocytes in response to LPS we conducted double immunofluorescence staining for GFAP and p65 as indicated in figure 2. The figure shows images with remaining time points from Figure 2. **B:** Images show representative astrocytes with negative, high and low levels of nuclear p65 staining in GFAP positive cells. This criteria was use to analyze p65 nuclear localization in figure 2. Arrows indicate astrocyte nuclei.

**Supplementary Figure 3**

**A:** AEC or microglia-reconstituted AEC (+ Microglia) were incubated with LPS during 1 h in reduced-serum medium. To address microglia percentage in the experiment shown in Figure 3, we immunostained these cultures against IBA1. **B:** Graph shows percentage of IBA1 positive cells in the indicated conditions. **C:** Images corresponding to the red channel (p65) of the images shown in Fig. 3A (60 min). It is of note that, in some fields, astrocyte p65 activation appears in a “cluster-like” configuration.

**D:** Separate wells from the same AEC shown in Figure 3 (used for conditioned medium exposure) were exposed to LPS to analyze LPS response capacity after 1 h of treatment and GFAP/p65 double inmunostaining. **E:** Graph shows no significant changes in any treatment after comparing to it's control (no LPS) using a One sample T-test. n=6.
